# Hypermethylation of MAPK13 Promoter in Oesophageal Squamous Cell Carcinoma Is Associated with Loss of p38δ MAPK Expression

**DOI:** 10.3390/cancers7040881

**Published:** 2015-10-23

**Authors:** Carol O′Callaghan, Liam J. Fanning, Orla P. Barry

**Affiliations:** 1 Department of Pharmacology and Therapeutics, University College Cork, Room 3.89, Western Gateway Building, Western Road, Cork, Ireland; cmocallaghan@gmail.com; 2 Molecular Virology Diagnostic and Research Laboratory, Department of Medicine, University College Cork and Cork University Hospital, Clinical Sciences Building, Cork, Ireland; l.fanning@ucc.ie

**Keywords:** p38δ MAPK, epigenetics, methylation, oesophageal squamous cell carcinoma

## Abstract

The loss of tumour suppressor gene function is a hallmark of malignant transformation and can occur by a variety of genetic and/or epigenetic alterations. We have previously characterised p38δ mitogen-activated protein kinase (MAPK) as a tumour suppressor in oesophageal squamous cell carcinoma (OESCC) and outlined how loss of p38δ MAPK expression promotes increased proliferation and migration, as well as reduced chemosensitivity. Our aim was to investigate the underlying molecular causes of loss of p38δ MAPK expression in OESCC. Sequence analysis of DNA from p38δ MAPK positive and p38δ MAPK negative OESCC cell lines was used to investigate potential loss of function causing mutations. Epigenetic control of p38δ expression in OESCC was examined using methylation-specific PCR and sequencing of bisulfite-converted DNA. We did not identify any mutations in the MAPK13 sequence in OESCC cell lines which lack p38δ MAPK expression. However, we identified a differential pattern of methylation between p38δ MAPK positive and p38δ MAPK negative cell lines. We outline here for the first time differential MAPK13 promoter methylation in OESCC. Our results suggest that epigenetic alterations are responsible, in part, for the suppression of p38δ MAPK expression and promotion of tumourigenesis in OESCC.

## 1. Introduction

We have previously documented the differential expression of p38δ mitogen activated protein kinase (MAPK) in oesophageal squamous cell carcinoma (OESCC) and the consequences of p38δ MAPK expression on OESCC tumourigenicity and response to cytotoxic drugs. Loss of p38δ MAPK expression contributes to a pro-oncogenic phenotype in OESCC and affords a level of resistance to conventional cisplatin and 5-fluorouracil treatment [1,2]. Re-introduction of p38δ and p-p38δ MAPK expression resulted in a decrease in OESCC cell proliferation, migration and anchorage-independent growth [1]. These results demonstrated significant tumour suppressive functions for p38δ MAPK in OESCC. Loss of p38δ MAPK expression can therefore be considered as a significant pro-survival mechanism in OESCC. In general, loss or inactivation of tumour suppressor genes occurs via genetic changes, such as point mutations and deletions or by epigenetic regulation [3]. This new study sheds light on some mechanism(s) by which the loss of p38δ MAPK expression may occur in OESCC.

At the genomic level, point mutations, insertions, and deletions of genetic material are common mechanisms by which expression of tumour suppressor genes are inactivated. The somatic mutation rate for OESCC is higher than in breast carcinoma, with C.G → T.A transitions the most common, followed by C.G → G.C transversions [4,5]. Point mutations can be responsible for converting a codon into a premature stop codon or replacing an essential amino acid thereby abolishing or reducing the production of a functioning gene product. Point mutations can also cause aberrant RNA splicing, again abolishing the function of the gene [6]. The tumour suppressor p53 is frequently mutated in OESCC and p53 mutated oesophageal cancer is recognised as being resistant to conventional chemotherapy [7,8]. Insertion or deletion of a single nucleotide can cause a frameshift in the coding sequence which has not been explored to date as a means for explaining loss of p38δ MAPK protein expression.

Cancer genomes are also characterised by epigenetic changes such as aberrant DNA methylation, in addition to mutations and chromosomal abnormalities [9,10,11]. DNA methylation occurs in cytosines which precede guanines *i.e.*, CpG dinucleotides and is essential for normal maintenance of tissue-specific gene expression [12]. In comparison with normal cells, cancer cells display global DNA hypomethylation, particularly in repetitive DNA sequences and introns which mediates genome instability [13,14,15]. This is coupled with specific hypermethylation of tumour suppressor genes which is responsible for gene silencing as DNA methylation inhibits transcription factor binding to promoter regions [16]. Hypermethylation of CpG-rich regions in gene promoters (known as CpG islands) is in fact one of the most common epigenetic changes to occur in tumours. Interestingly, it is found in almost all types of human neoplasms and gene inactivation by epigenetic silencing is at least as common as the classic coding-region mutation based disruption of tumour suppressor genes [17,18,19,20]. This form of epigenetic regulation is increasingly implicated in the development and progression of many cancers, including oesophageal carcinomas where tumour suppressor genes involved in the cell cycle, such as p16^INK4b^ and p14^ARF^, are frequently hypermethylated [3,16]. Notably, promoter hypermethylation has previously been implicated in the epigenetic silencing of p38δ MAPK in different cancer types. In malignant pleural mesothelioma and primary cutaneous melanoma MAPK13 promoter methylation is associated with decreased p38δ MAPK mRNA and protein expression [21,22]. Restoration of p38δ MAPK expression in melanoma cells decreases proliferation [21].

In this study mRNA and gDNA sequences from p38δ MAPK protein positive and negative cell lines were compared in order to identify any of the genetic abnormalities discussed above, which may explain the differential loss of p38δ MAPK expression. Possible epigenetic regulation of p38δ MAPK expression was investigated using methylation-specific PCR (MSP) and bisulfite sequencing PCR (BSP) analysis of CpG islands in the MAPK13 promoter region.

## 2. Results and Discussion

### 2.1. OESCC Cell Lines Lacking Endogenous p38δ MAPK Expression Proliferate and Migrate Faster than Those Which Express This Isoform

**Figure 1:**
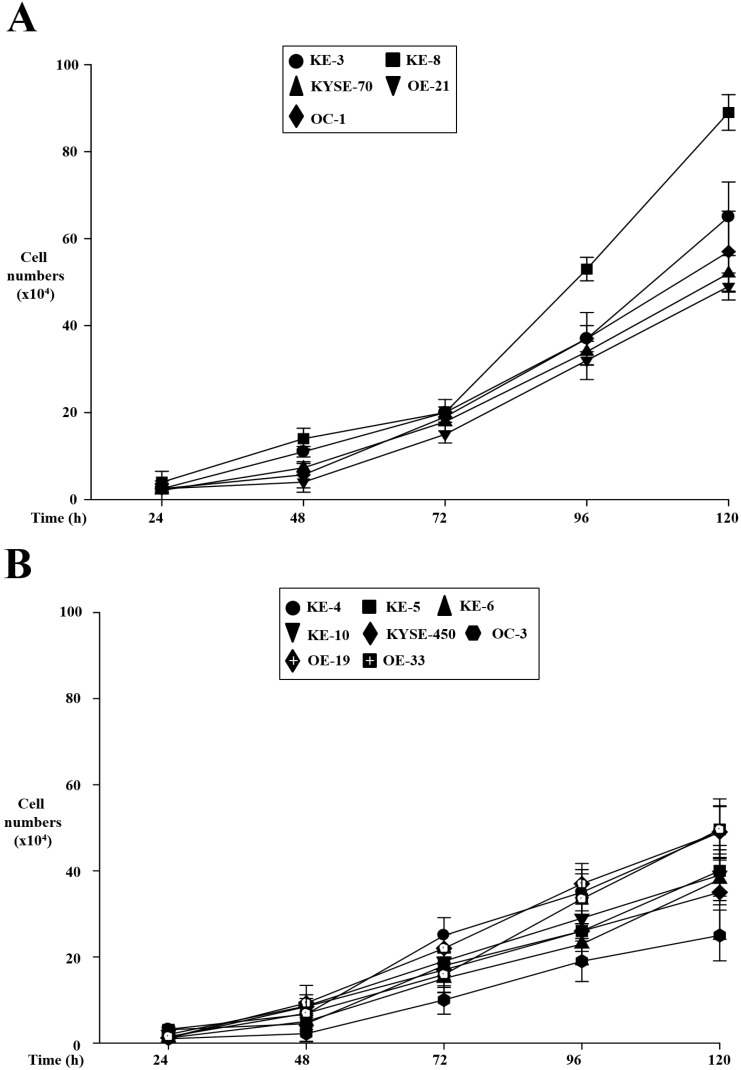

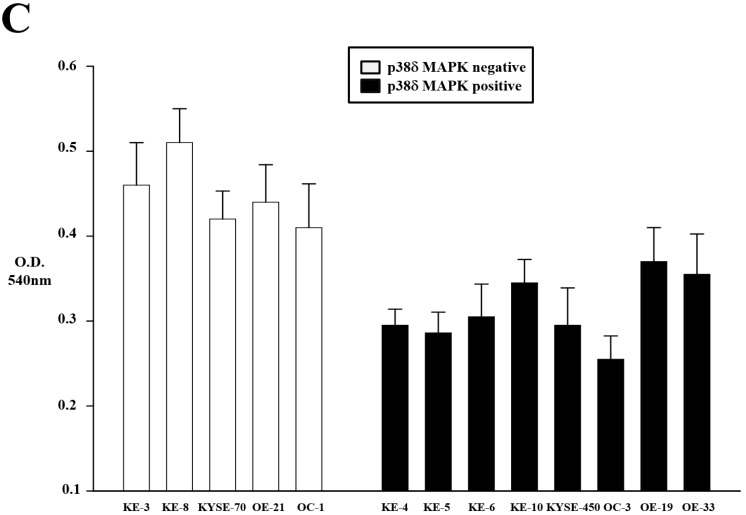
Effect of p38δ MAPK status on oesophageal cancer cell growth and migration. Oesophageal p38**δ** MAPK negative (KE-3 and -8, KYSE-70, OE-21, and OC-1) (**A**) and positive (KE-4, -5, -6, and -10, KYSE-450, OC-3, OE-19, -21 and -33) (**B**) cell lines were examined for their cell proliferation rate. Cells were seeded (3 × 10^4^) in six-well plates and counted for 24–120 h (**A**,**B**); (**C**) Effect of p38**δ** on cell migration using a Boyden Chamber as described in the Experimental Section. The results shown are mean ± S.E. of three independent experiments.

We have previously identified that p38δ MAPK plays a key role in OESCC cell proliferation and migration using a small number of cell lines [1]. We have now extended these earlier reports using a larger number of cell lines lacking p38δ MAPK expression (KE-3 and -8, KYSE-70, OE-21, and OC-1) as well as those that express p38δ MAPK (KE-4, -5, -6 and -10, KYSE-450, OC-3, OE-19, -21, and -33). Using the trypan blue exclusion assay we compared the proliferation rate of p38δ negative and positive oesophageal cell lines. We observed that at all time-points studied (24–120 h) the p38δ negative OESCC cell lines grew significantly (*p* < 0.01) faster than the p38δ positive cell lines (Figure 1A,B). Of note, the two p38δ positive OE-19 and OE-33 are oesophageal adenocarcinoma cell lines and did not demonstrate a statistically significant difference in their proliferation compared to the p38δ negative OESCC cell lines. We have previously reported that differential p38δ MAPK expression is only observed in OESCC and not adenocarcinoma cell lines [1]. A key characteristic of cancer cells is their ability to migrate and progress from primary tumours to metastases in distant organs. We examined whether the p38δ status of OESCCs could influence their rate of cell migration. Using a Boyden Chamber assay and allowing the cells to migrate for 24 h we observed that all five p38δ negative OESCC cells migrated significantly (*p* < 0.01) faster than their p38δ positive counterparts (Figure 1*C*).

### 2.2. p38δ MAPK mRNA Is Present in All OESCC Cell Lines

**Figure 2:**
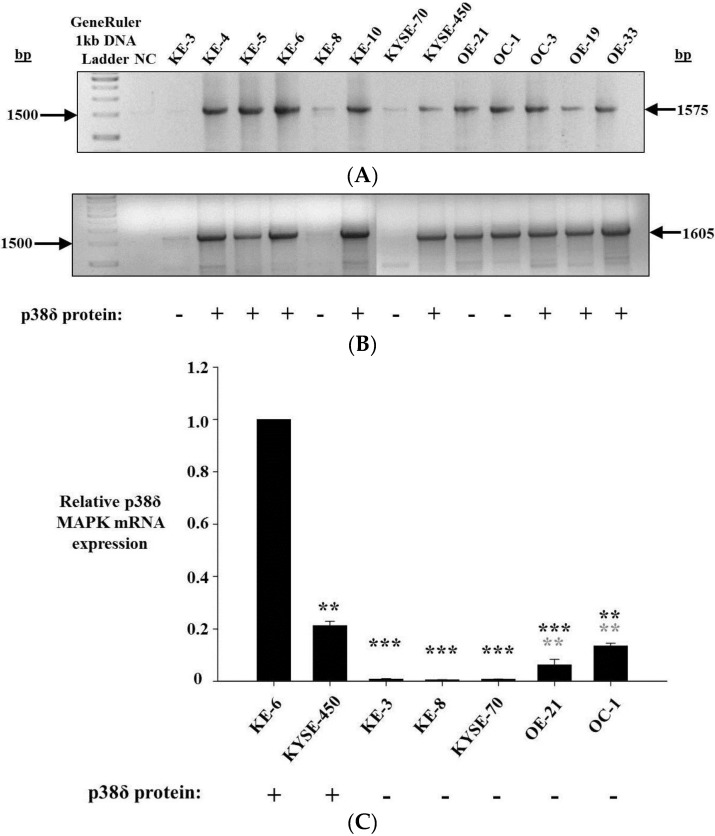
p38δ MAPK mRNA expression in oesophageal cancer. (**A**,**B**) Agarose gel electrophoresis analysis of DNA fragments produced by PCR amplification of p38δ MAPK mRNA from p38δ MAPK protein positive (KE-4, KE-5, KE-6, KE-10, KYSE-450, OC-3, OE-19, OE-33) and p38δ MAPK protein negative (KE-3, KE-8, KYSE-70, OE-21, OC-1) oesophageal carcinoma cells with (**A**) P002F and P001R primers and (**B**) P003F and P001R primers; and (**C**) relative p38δ MAPK mRNA levels in p38δ MAPK protein negative OESCC cell lines KE-3, KE-8, KYSE-70, OE-21 and OC-1 were compared with p38δ MAPK protein positive KE-6 and KYSE-450 cells. GAPDH was measured as an internal control. Relative values were calculated by normalizing 2^−ΔΔCT^. Results shown are the mean ± S.E. of three independent qrt-PCR experiments. *** *p* < 0.001, ** *p* < 0.01, significant changes in p38δ MAPK expression from (black) KE-6 and (grey) KYSE-450 cells were determined by application of Student′s *t*-test.

Intron-spanning primer sets within the p38δ MAPK coding region amplified cDNA from p38δ MAPK protein positive cell lines KE-4, -5, -6 and -10, KYSE-450, OC-3, OE-19, and -33, as well as from p38δ MAPK protein negative cell lines OE-21 and OC-1 (Figure 2A,B). Varying levels of amplicon were observed from KE-3, KE-8, and KYSE-70 (p38δ MAPK protein negative) cells, depending on the primer pair used. Primers specific for a 1575 bp portion of p38δ mRNA (P002F and P001R) weakly amplified cDNA from KE-8 and KYSE-70 cells, but not from KE-3 cells (Figure 2A). PCR with a primer pair specific for a 1605 bp region of p38δ mRNA (P003F and P001R) did not yield any detectable product from KYSE-70 cDNA but did weakly amplify cDNA from KE-3 and KE-8 cells (Figure 2B). In the absence of any detectable mutations at the genomic level, differential p38δ MAPK mRNA expression was the most likely cause of p38δ MAPK protein loss. Therefore, p38δ MAPK mRNA expression was further analysed. Quantitive real-time PCR was used to determine the relative levels of p38δ MAPK mRNA expression in each of the OESCC cell lines being studied. p38δ MAPK mRNA expression was found to be highest in p38δ MAPK protein positive cells KE-6 and KYSE-450, although there was a significant (*p* < 0.01) difference between the two cell lines (Figure 2C). p38δ MAPK mRNA expression was similarily and significantly (*p* < 0.001) down-regulated in p38δ MAPK protein negative cell lines KE-3, KE-8, and KYSE-70 when compared with both KE-6 and KYSE-450 p38δ MAPK protein positive cells (Figure 2C). OE-21 and OC-1 (both p38δ MAPK protein negative) demonstrated intermediate levels of p38δ MAPK mRNA expression yet still with significant (*** *p* < 0.001 and ** *p* < 0.01) decreases from KE-6 and KYSE-450 expression (Figure 2C).

### 2.3. MAPK13 Promoter Is Differentially Methylated in OESCC

**Figure 3:**
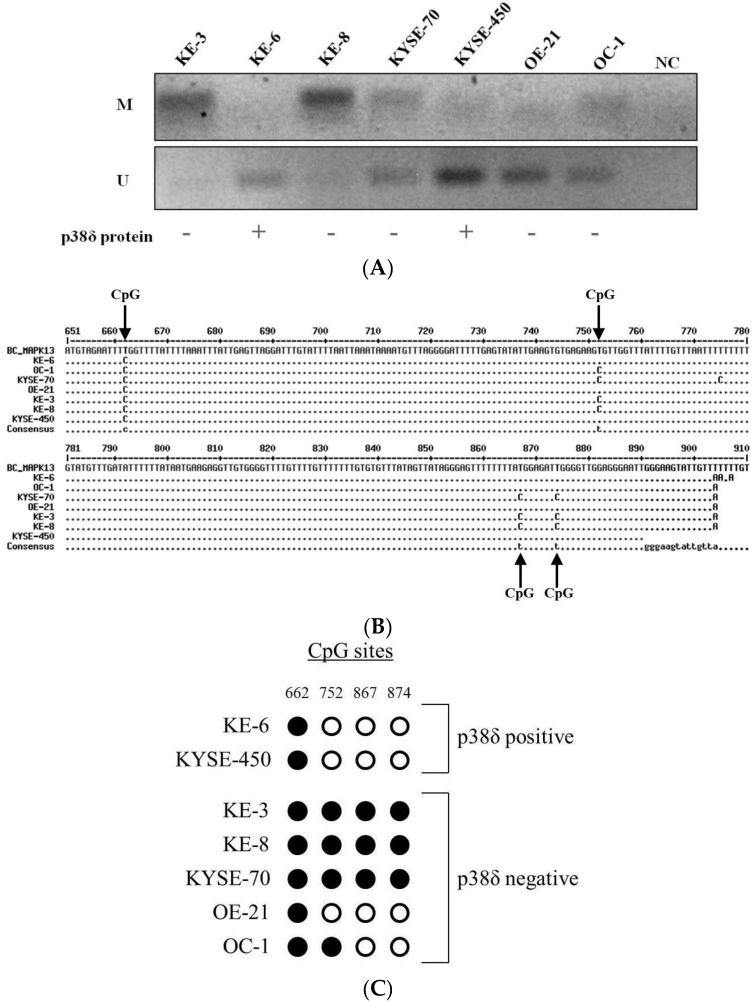
MAPK13 promoter methylation analysis. (**A**) Agarose gel electrophoresis analysis of MAPK13 (Methylation-Specific PCR) MSP products (p38δ MAPK protein positive cell lines, KE-6, KYSE-450 and p38δ MAPK protein negative cell lines, KE-3, KE-8, KYSE-70, OE-21, OC-1; (**B**) DNA sequence analysis of MAPK13 CpG island Bisulfite Sequencing PCR (BSP) products. Residues identical to the reference sequence (MAPK13 NC_000006.12) residue at the same position are represented by a point (•), residues which differ are in uppercase; and (**C**) representative BSP analysis of CpG methylation. An unmethylated cytosine is depicted as a white circle, a methylated cytosine as a black circle.

As a genetic mutation had been ruled out as an explanation for the differential loss of p38δ MAPK in OESCC, epigenetic regulation of p38δ MAPK expression was considered. DNA methylation was detected by methylation-specific PCR (MSP) in KE-3, KE-8, KYSE-70, and OC-1 cells (Figure 3A (M)). In contrast, no substantial DNA methylation was identified in KE-6, KYSE-450, and OE-21 cells (Figure 3A (M)). Furthermore, unmethylated DNA was poorly amplified in KE-3, KE-8, and KYSE-70 cells (Figure 3A (U)). This is indicative of the presence of MAPK13 promoter hypermethylation in p38δ MAPK protein negative OESCC (with the exception of the OE-21 cell line).

Bisulfite sequencing PCR (BSP) was used for further validation and analysis of MAPK13 promoter methylation density in OESCC. The methylation status of four individual CpG sites within a MAPK13 promoter region CpG island was analysed. Comparison of DNA sequence reads of PCR products with in silico bisulfite-converted MAPK13 reference sequence (NC_000006.12) identified differential CpG methylation in OESCC (Figure 3B). Unmethylated CpG cytosines (C) in the MAPK13 reference sequence are converted to uracil (T). Identical residues at the equivalent positions in the PCR products indicates the CpG residue is not methylated in that particular cell line while the presence of unconverted C residues is indicative of DNA methylation at that individual CpG site (Figure 3B).

Of the four MAPK13 promoter region CpG sites analysed by BSP, only one was found to be methylated in KE-6 and KYSE-450 p38δ MAPK protein positive OESCCs (Figure 3B,C). Conversely, DNA methylation was detected at all four CpG sites in KE-3, KE-8, and KYSE-70 p38δ MAPK protein negative OESCCs (Figure 3B,C). In p38δ MAPK protein negative OE-21 and OC-1, DNA methylation was identified at one and two CpG sites respectively (Figure 3B,C).

## 3. Experimental Section

***Cell culture***- The KE oesophageal cancer cell lines (kind gifts from Professor T. Fujii, Kurume University School of Medicine, Japan) [23,24,25] as well as KYSE-70, OE-19, OE-21, and OE-33 (ATCC, Rockville, MD, USA) were cultured in RPMI-1640 supplemented with 10% FCS, 100 µg/mL streptomycin and 100 units/mL penicillin. KE cell line features have previously been summarized by us [1]. OC-1 and OC-3 (kind gifts from Cork Cancer Research Centre, (Biosciences Institute, National University of Ireland, Cork, Ireland) [26] were cultured in DMEM supplemented with 10% FCS, 100 µg/mL streptomycin and 100 units/ml penicillin. KYSE-450 cells (ATCC, Rockville, MD, USA) were maintained in 45% RPMI-1640/45% Ham′s F-12 Nutrient Mixture supplemented with 10% FCS, 100 µg/mL streptomycin and 100 units/mL penicillin.

***Proliferation assay***—Cells were plated at a density of 3 × 10^4^ cells/well in a six-well tissue culture plate. Cell viability was assessed by trypan blue (0.4% w/v) exclusion assay at the indicated times [26].

***Boyden chamber cell migration assay****—*Cells were plated in starvation medium at a density of 3 × 10^4^ cells/well into a 96-well plate of the upper chamber. The bottom chamber contained 10% FCS as the chemoattractant. Cells were left migrate for 24 h through the matrigel filter (8 mm). Migrated cells were treated with MTT (3-(4,5-dimethylthiazol-2-yl)-2,5-diphenyltetrazolium bromide) (5 mg/mL) and absorbance read at 540 nm to calculate viable cell numbers as previously described [1].

***PCR***—1575bp and 1605bp fragments of p38δ MAPK mRNA, corresponding to positions 111–1686 and 82–1686 respectively of NCBI Reference Sequence NM_002754.4 were amplified from cellular cDNA using oligonucleotide primers P003F (5′-CGAGATCGGGTGCCCGGGAT-3′) or P002F (5′-CCGGAAAAAGGGCTTCTACAA-3′) and P001R (5′-CCGCCACAAGCTAAAAAGAG-3′).

***Quantitative rt-PCR***—p38δ MAPK mRNA expression was quantitated using a Qiagen QuantiFast^®^ MAPK13 Probe Assay with one-step rt-PCR and simultaneous detection of GAPDH according to manufacturer′s instructions.

***Methylation analysis****—*The methylation status of DNA was determined using sodium bisulfite conversion with an EpiMark^®^ Bisulfite Conversion Kit (New England BioLabs, Hertfordshire, UK) according to manufacturer′s instructions. Methylation-specific PCR (MSP) is a method of assessing the methylation status of a group of CpG sites using individual pairs of primers specific for methylated (M) *versus* unmethylated (U) DNA [27]. MSP primers (Table 1) were designed within a CpG island in the MAPK13 promoter region (NC_000006.12 36130157-36130872) with the aid of MethPrimer, an online program for designing bisulfite-conversion-based primers [28]. For bisulfite sequencing PCR (BSP) oligonucleotide primers 5′-TTGGGAGTTGGTTAGAAATGTA-3′ and 5′-AACAATACTTCCCAATTCCCT-3′ were designed to amplify a 270 bp fragment from bisulfite converted DNA. PCR products were analysed by DNA sequencing to determine the average methylation status for each of the four CpG dinucleotides present in the amplicon.

**Table 1:** Primers for Methylation-Specific PCR (MSP).

	Primer	Sequence (5′→3′)
**M**	Forward	TTTGTTTGGATTTATTAGTTTCGTC
	Reverse	GAACCTATCCAACCCTACGCT
**U**	Forward	GTTTGGATTTATTAGTTTTGTTGT
	Reverse	CAAACCTATCCAACCCTACACT

## 4. Conclusions

Tumour suppressor gene function can be lost in a variety of ways in order for cancer cells to gain the capacity for uncontrolled proliferation, migration, and escape from apoptosis. These include somatic mutations, including insertions, deletions or point mutations, and/or epigenetic changes such as DNA methylation or histone deacetylation. p38δ MAPK displays tumour suppressor functions in OESCC—its loss promotes proliferation, migration, anchorage-independent growth, and resistance to conventional chemotherapy [1,2]. p38δ MAPK expression is absent at the protein level in OESCC cell lines KE-3, KE-8, KYSE-70, OE-21, and OC-1. In this study we outlined how this inactivation of p38δ MAPK expression may occur in OESCC.

The results presented here indicate that this loss of protein expression cannot be attributed to a genetic mutation or deletion. p38δ MAPK mRNA is expressed in all OESCC cell lines, regardless of p38δ MAPK protein status. However, differential mRNA expression levels were observed between p38δ MAPK protein positive and protein negative cell lines. The highest levels of p38δ MAPK mRNA expression were observed in p38δ protein positive cell lines KE-6 and KYSE-450. Significantly lower levels of p38δ MAPK mRNA were detected in p38δ MAPK protein negative cell lines. This suggests that the absence of p38δ MAPK protein expression in these cells may be attributed to p38δ MAPK mRNA expression failing to reach the threshold required to achieve a detectable level of protein synthesis.

Our results show that the downregulation of p38δ MAPK mRNA expression in KE-3, KE-8, KYSE-70, and OC-1 cells is most likely associated with MAPK13 promoter hypermethylation. The MAPK13 promoter regions of the OESCC cell lines with the lowest levels of p38δ MAPK mRNA expression (KE-3, KE-8, and KYSE-70 p38δ protein negative cells) are highly methylated, as demonstrated by both MSP and BSP analysis. Conversely, in p38δ MAPK protein positive cell lines KE-6 and KYSE-450, in which p38δ MAPK mRNA expression was significantly higher (*** *p* < 0.001), a decreased incidence of DNA methylation was detected by both MSP and BSP. OC-1 cells also expressed significantly higher (*** *p* < 0.001) levels of p38δ MAPK mRNA than KE-3, KE-8, and KYSE-70 cells despite the fact that they are also p38δ MAPK protein negative. Interestingly, however, the MAPK13 promoter was also found to be hypermethylated in this cell line when compared with p38δ MAPK protein positive cells (two CpG sites methylated *versus* one CpG site methylated). The significance of these results is that there appears to be an inverse correlation therefore between MAPK13 promoter methylation and p38δ MAPK mRNA expression in OESCC. Of note, methylation of the specific CpG sites examined here may not be the critical determinants of MAPK13 expression but rather these results reflect a general pattern of MAPK13 promoter methylation. This would explain why the OE21 cell line does not appear to fit this model of p38δ MAPK epigenetic regulation—the loss of p38δ MAPK expression in the OE21 cell line may be caused by MAPK13 promoter methylation at a critical CpG site not included in this analysis.

The implication of MAPK13 promoter methylation in promoting tumourigenesis in OESCC could have therapeutic inferences. Epigenetic alterations, as opposed to genetic mutations can be pharmacologically manipulated. The DNA methyltransferase inhibitor 5′-aza-2′-deoxycitidine (as decitabine) is used as a treatment for myelodysplastic syndromes and leukaemia [29,30]. Potential lies in the combination of epigenetic and non-epigenetic therapies. The re-expression of tumour suppressor genes by epigenetic drugs weakens the ability of cancer cells to withstand cytotoxic treatment, thereby lowering the dose of chemotherapeutic drug required to induce cell death [31]. Another potential clinical use of the findings presented here is as a prognostic tool. DNA methylation analysis techniques would facilitate the sensitive and quantitative detection of a hypermethylated MAPK13 promoter in biopsy specimens. Pre-treatment identification of p38δ MAPK status may be useful in determining the optimal treatment and predicting response in OESCC patients.

In conclusion, promoter hypermethylation of the MAPK13 gene in KE-3, KE-8, KYSE-70, and OC-1 OESCC cells inhibits transcription of p38δ MAPK, resulting in down-regulation of p38δ MAPK mRNA expression. This decrease in p38δ mRNA correlates with a loss of p38δ MAPK protein expression that is associated with increased proliferation and migration in OESCC.
